# The resistive switching memory of CoFe_2_O_4_ thin film using nanoporous alumina template

**DOI:** 10.1186/1556-276X-9-584

**Published:** 2014-10-21

**Authors:** Changjun Jiang, Lei Wu, WenWen Wei, Chunhui Dong, Jinli Yao

**Affiliations:** 1 Key Lab for Magnetism and Magnetic Materials of the Ministry of Education, Lanzhou University, Lanzhou 730000, People's Republic of China

**Keywords:** Nanowire, Thin film, Electrochemical deposition, Resistive random access memory

## Abstract

A novel conductive process for resistive random access memory cells is investigated based on nanoporous anodized aluminum oxide template. Bipolar resistive switching characteristic is clearly observed in CoFe_2_O_4_ thin film. Stable and repeatable resistive switching behavior is acquired at the same time. On the basis of conductive filament model, possible generation mechanisms for the resistive switching behaviors are discussed intensively. Besides, the magnetic properties of samples (before and after the annealing process) are characterized, and the distinct changes of magnetic anisotropy and coercive field are detected. The present results provide a new perspective to comprehend the underlying physical origin of the resistive switching effect.

**PACS:**

68.37.-d; 73.40.Rw; 73.61.-r

## Background

The high-performance nonvolatile memory is greatly demanded in modern information technology. Resistive random access memory (RRAM) is a promising candidate among the emerging nonvolatile memory technologies [1-3]. Compared with magnetic random access memory (MRAM), the important attributes of RRAM were capacitor-like cell structure, ultrafast operating speeds, high scalability, and low power consumption, which allowed it to have more superiorities in application. There have been active researches on the scaling of resistive switching (RS) memory devices. At first, RS effect has been widely investigated in numerous binary transition oxides such as ZnO, TiO_2_, and NiO [4-6]. Recently, various ferrites (NiFe_2_O_4_, CoFe_2_O_4_) [7-9] and multiferroic materials (BiFeO_3_) [10,11] are both found to exhibit RS behavior. Cobalt ferrite, due to its rich and unique magnetic and electronic behaviors such as magneto-optic effect and magnetoelectric effect [12], is extensively investigated. Meanwhile, it has been considered as an important component in multilayers or composites for multiferroic research and application [13]. However, the previous studies of RS behaviors are mainly focused on thin film structures, and the underlying physical origin of the RS effect is still a controversial issue. Thus, it is of significant importance to explore new RS structures and elucidate the RS physical mechanism.

Notably, lower dimension is beneficial to illustrate the nature of material, and ordered arrays of isolated nanostructures are of considerable to elucidate the RS physical mechanism. Typically, various lithographic techniques have been used to fabricate regular arrays of nanostructures, such as electron-beam lithography and focused ion beam technology, [14-16] but high production cost and long processing time are needed. Relatively, nanoporous anodized aluminum oxide (AAO) have been widely used as the mask for the fabrication of uniform nanoscale patterns because nanoscale materials/devices can be easily synthesized through electro-deposition or physical vapor deposition [17,18].

In this paper, we demonstrate a novel conductive process for resistive random access memory cells based on nanoporous AAO filled with CoFe nanowires and covered by a layer of CoFe_2_O_4_ film. The magnetic properties of samples (before and after the annealing process) are characterized. Bipolar resistive switching characteristic is clearly observed in our sample. On the basis of conductive filament model, possible generation mechanisms for the resistive switching behaviors are discussed intensively.

## Methods

Nanoporous AAO template is fabricated by a two-steps oxidization process [19]. An ordered porous alumina layer containing straight, parallel pores with an average diameter of 50 nm is prepared. The electrodeposition of the FeCo alloy nanowire arrays and films is performed by using a standard double electrode bath. The AAO template is used as one electrode, and the graphite is used as another. The electrolyte contains FeSO_4_ · 7H_2_O (30 g/l), CoSO_4_ · 7H_2_O (17.9 g/l), and H_3_BO_4_ (10 g/l). The pH value of the electrolyte is maintained at about 3.0, and the AC electrodepositions are conducted at 200 Hz and 15 V for 1 h. Due to deposition after long time, CoFe alloy film is formed at AAO surface and connected with nanowires. Then, the sample is annealed in the air, which induces a spinel oxide layer CoFe_2_O_4_ (cobalt ferrite (CFO)) at the surface. Au dots are sputtered on the top of CoFe_2_O_4_ as electrodes by magnetron sputtering using mask at room temperature. Cu wires are connected to the electrodes by adhesive tape. The as-synthesized samples were characterized by X-ray diffractometer (XRD; X' Pert PRO PHILIPS with Cu Kα radiation, *λ* =1.54056 Å). The morphologies of the samples were characterized by scanning electron microscopy (SEM; Hitachi S4800, Hitachi, Ltd., Chiyoda-ku, Japan). Magnetic properties of the samples at room temperature (RT) were measured by using the vibrating sample magnetometer (MicroSense VSM EV9, MicroSense, Massachusetts, USA). The electrical properties of CFO thin films were tested in the air using a Keithley 2400 source measurement unit (Keithley 2400, Keithley Instruments Inc., Cleveland, USA). All of the tests were obtained at room temperature.

## Results and discussion

**Figure 1:**
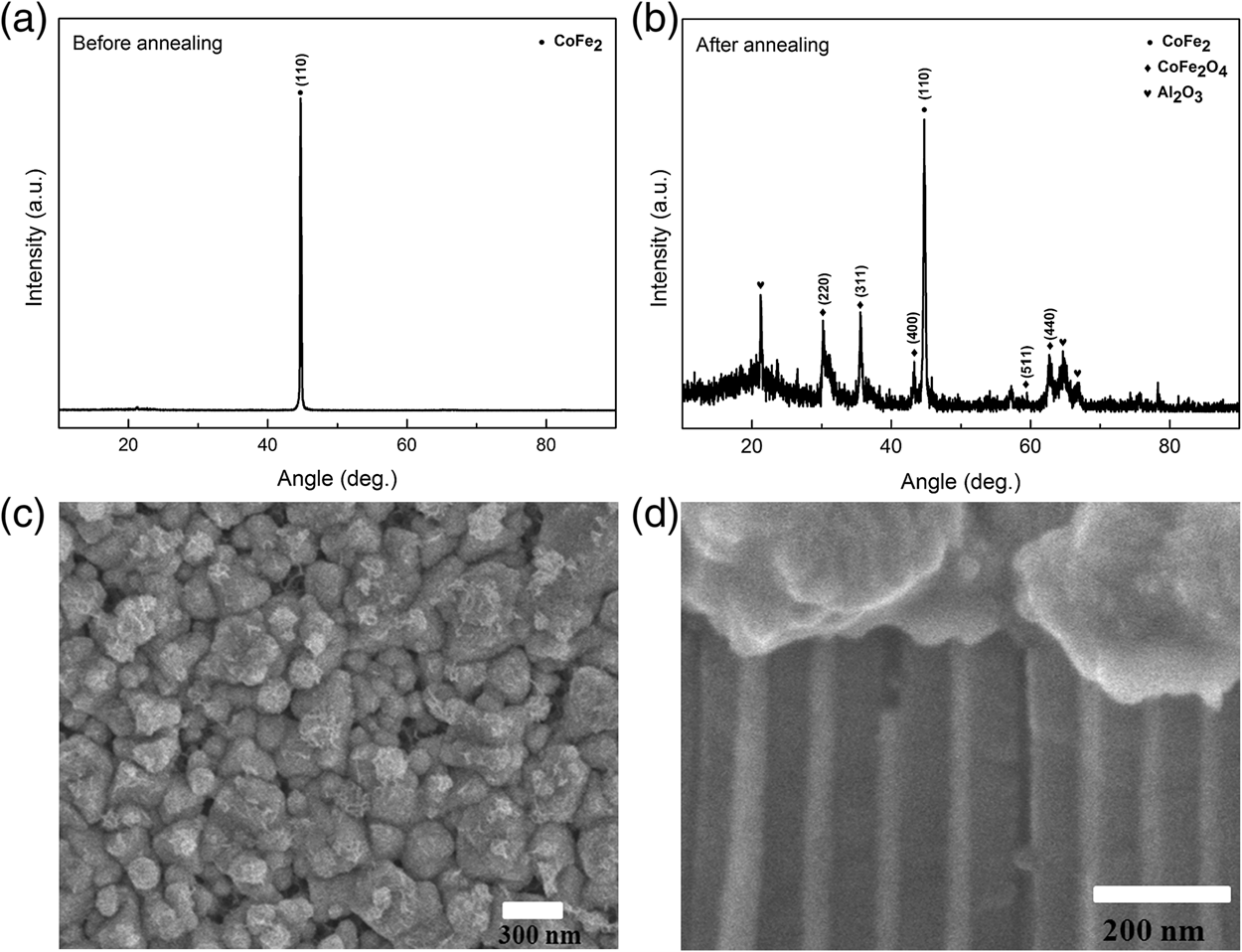
**The XRD pattern of sample before and after annealing and SEM images. (a,b)** the XRD pattern of sample before and after annealing and SEM images of sample in **(c)** top-views and **(d)** cross-sectional views.

Figure 1a shows X-ray diffraction (XRD) pattern of the sample before annealing and indicates that pure CoFe_2_ alloy phase is detected. After annealing at 500 °C in the air, the surface and parts of nanowire CoFe_2_ are oxidized to spinel phase CoFe_2_O_4_, as shown in Figure 1b. Figure 1c,d displays the SEM images of CoFe_2_-filled AAO membrane in top-views and cross-sectional views, respectively. It becomes clear that the membranes are continuous embedded nanowires in the pores, and a layer of thin film is formed on the surface of AAO template. The diameters of the nanowires are about 50 nm. Moreover, SEM images indicated the connection between nanowires and film.

**Figure 2:**
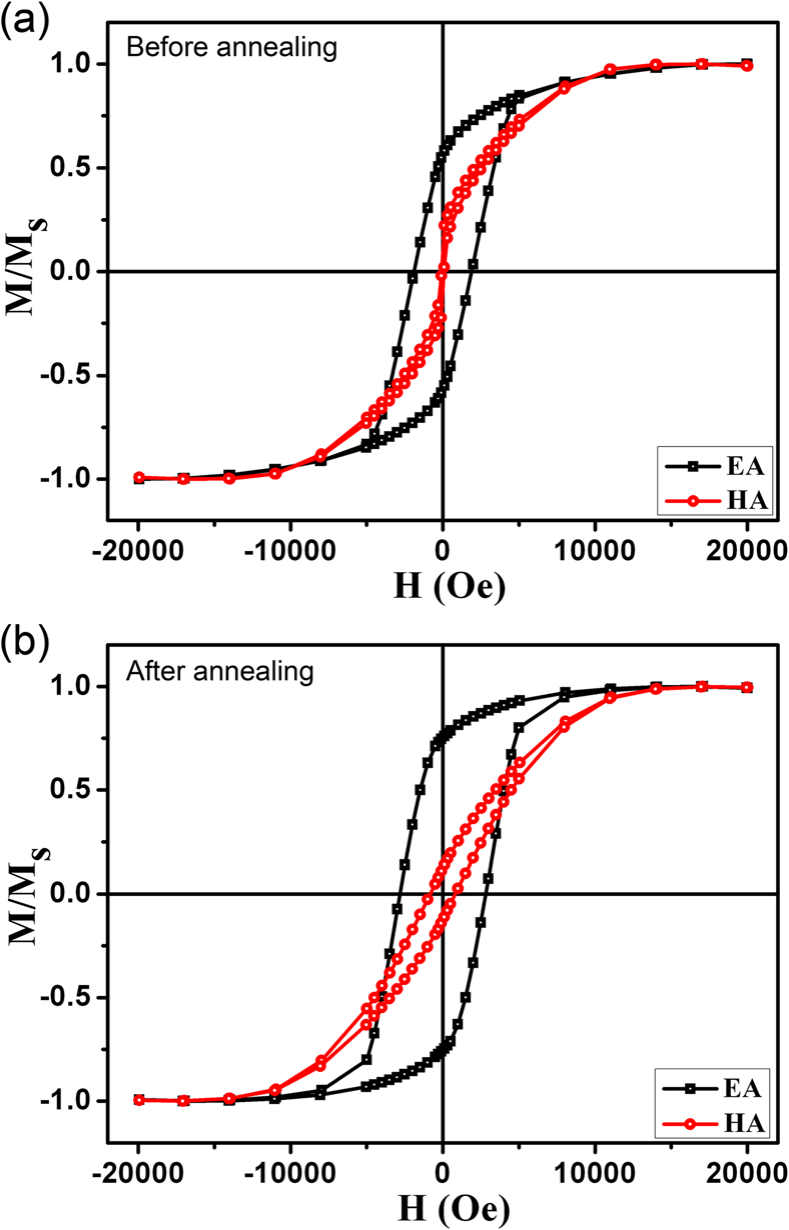
**Magnetic hysteresis loops of the sample before and after annealing. (a,b)** Magnetic hysteresis loops of the sample within AAO measured with the applied field parallel and perpendicular to the axis of the nanowires at room temperature before and after annealing, respectively.

The *M*-*H* curves at RT for the samples before and after annealing are presented in Figure 2. The black and red lines in the figures represent hysteresis loops with external magnetic field parallel and perpendicular, respectively. Along the direction of nanowire axis, obvious shape anisotropy was displayed. Compared with pure CoFe film, the coercive fields are distinctly enhanced after annealing. This is attributed to pinning effect due to oxide of nanowires surface. Note that the decrease of remnant magnetization along perpendicular to nanowire axis after annealing was compared with that of before annealing, which resulted from oxide of CoFe alloy.

**Figure 3:**
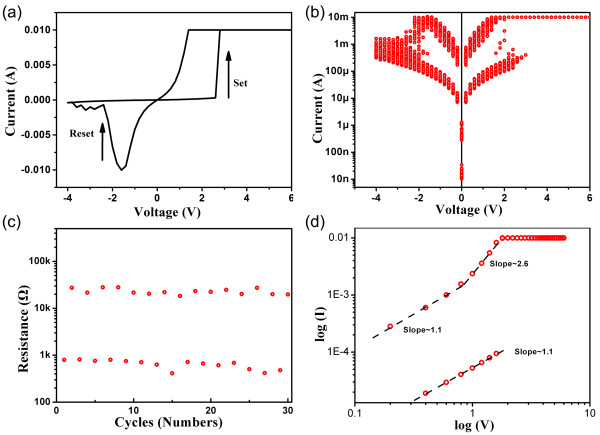
**The characteristics of sample and the *I*-*V *** **curve (a) the *I-V *** **characteristics of sample, the *I*-*V *** **curve in (b) semi-log scale and (d) double-log scale, and (c) the resistances versus pulse voltage in semi-log scale.**

**Figure 4:**
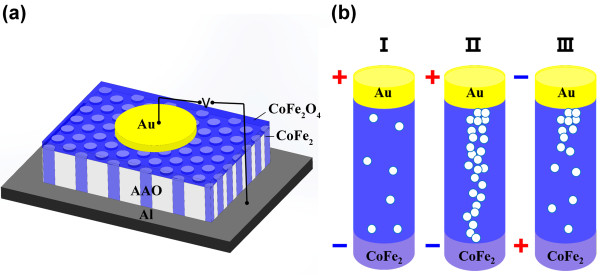
**The schematic illustration of sample and the scheme of operation of a memory cell. (a)** the schematic illustration of sample and **(b)** the scheme of operation of a memory cell based on conductive filament model.

The current-voltage (*I*-*V*) cycle of the sample after annealing is plotted in Figure 3a. The bias voltage is swept as 0 V → 6 V → 0 V → -4 V → 0 V, the bipolar RS characteristics are obtained. As the positive voltage increased at around 2.5 V, the current increased suddenly, which indicated that the cell switched from high-resistance states (HRS) to low-resistance states (LRS), it has been defined as set process. When the negative voltage was applied on the device, the switching from LRS to HRS was occurred at about -1.5 V (defined as reset process). The schematic diagram of the structure was displayed in Figure 4a, Au top electrodes with diameter of 0.1 mm were deposited on the films, and the bottom of the sample is Al substrate. The positive voltage is defined as form top to bottom. Figure 3b depicts the *I*-*V* curve in semi-log scale for repeat measurements, good repeatability is exhibited. To investigate the stability of RS behaviors, the resistances changing with pulse numbers are displayed in Figure 3c, the stable variation is also presented.

In order to get further insight into the underlying mechanism on RS behavior, the double-log plots of *I-V* curves are studied in Figure 3d, which come from positive parts of Figure 3a. At low voltage, the *I-V* characteristics of the positive bias in both HRS and LRS present a linearly Ohmic behavior with a slope of 1 (*I* ∝ *V*). Then, there is a steep current increase region with the increase of voltage in LRS, a slope of about 2.6 is observed, which is corresponding to the trap-controlled space charge limited current (SCLC) model [20].

Since the unique structure of sample, current conduction is separated along each nanowire branch after flowing across the CoFe_2_O_4_ film, which eventually realizes RS behavior on nanoscale. On the basis of the aforementioned experimental results, the conductive filament models are applicative in our samples. The sketches of formation and rupture of oxygen vacancies (OVs) in Au/CoFe_2_O_4_/CoFe_2_ memory devices can be depicted in Figure 4b. When applied a positive voltage, the ionized OVs migrate towards the cathode and accumulate at CoFe_2_O_4_/CoFe_2_ interface, these OVs capture the electrons injected from the cathode, and cause the Fe^3+^ in the oxygen-deficient region reduced to Fe^2+^. The Fe^2+^ and the OVs can form a nonstoichiometric and highly conducting phase [21]. This highly conducting phase starts to create at the cathode and extends to the anode. Then, with the increase of voltage, a metallically conductive path is built, and the memory cell is switched to LRS, as displayed in Figure 4b II. The state is kept until a sufficient opposite voltage is applied. The negative bias can release the electrons from the neutral OVs, then the conductive filaments are dissolved, and the memory cell is switched to HRS as shown in Figure 4b III.

## Conclusions

In summary, a novel conductive process for resistive random access memory cells is investigated based on nanoporous anodized aluminum oxide template, which eventually realizes RS behavior on nanoscale. Stable and repeatable RS behavior is clearly observed. On the basis of conductive filament model, possible generation mechanisms for the resistive switching behaviors are discussed intensively. The present results provide a new perspective to comprehend the underlying physical origin of the resistive switching effect.

## Competing interests

The authors declare that they have no competing interests.

## Authors’ contributions

WW fabricated the samples and performed the measurements. LW and CJ analyzed the results and wrote the manuscript. CD and JY helped to measure the films and analyze the results. All authors read and approved the final manuscript.

## References

[B1] Waser R Dittmann R Staikov G Szot K Redox-based resistive switching memories - nanoionic mechanisms, prospects, and challenges Adv Mater 2009 21 2632 2663 10.1002/adma.200900375 36751064

[B2] Yang CH Seidel J Kim SY Rossen PB Yu P Gajek M Chu YH Martin LW Holcomb MB He Q Maksymovych P Balke N Kalinin SV Baddorf AP Basu SR Scullin ML Ramesh R Electric modulation of conduction in multiferroic Ca-doped BiFeO_3_ films Nature Mater 2009 8 485 493 10.1038/nmat2432 19396162

[B3] Yang Y Choi S Lu W Oxide heterostructure resistive memory Nano Lett 2013 13 2908 2915 10.1021/nl401287w 23724783

[B4] Chen G Song C Chen C Gao S Zeng F Pan F Resistive switching and magnetic modulation in cobalt-doped ZnO Adv Mater 2012 24 3515 3520 10.1002/adma.201201595 22678882

[B5] Choi BJ Jeong DS Kim SK Rohde C Choi S Oh JH Kim HJ Hwang CS Szot K Waser R Reichenberg B Tiedke S Resistive switching mechanism of TiO_2_ thin films grown by atomic-layer deposition J Appl Phys 2005 98 033715 10.1063/1.2001146

[B6] You YH So BS Hwang JH Cho W Lee SS Chung TM Kim CG An KS Impedance spectroscopy characterization of resistance switching NiO thin films prepared through atomic layer deposition Appl Phys Lett 2006 89 222105 10.1063/1.2392991

[B7] Hu W Qin N Wu G Lin Y Li S Bao D Opportunity of spinel ferrite materials in nonvolatile memory device applications based on their resistive switching performances J Am Chem Soc 2012 134 14658 14661 10.1021/ja305681n 22931305

[B8] Chhaya UV Mistry BV Bhavsar KH Gadhvi MR Lakhani VK Modi KB Joshi US Structural parameters and resistive switching phenomenon study on Cd_0.25_Co_0.75_Fe_2_O_4_ ferrite thin film Indian J Pure Appl Phys 2011 49 833

[B9] Hu W Zou L Chen R Xie W Chen X Qin N Li S Yang G Bao D Resistive switching properties and physical mechanism of cobalt ferrite thin films Appl Phys Lett 2014 104 143502 10.1063/1.4870627

[B10] Wu L Jiang C Xue D Resistive switching in doped BiFeO_3_ films J Appl Phys 2014 115 17D716 10.1063/1.4865217

[B11] Yan F Xing GZ Li L Low temperature dependent ferroelectric resistive switching in epitaxial BiFeO_3_ films Appl Phys Lett 2014 104 132904 10.1063/1.4870503

[B12] Huang W Zhu J Zeng HZ Wei XH Zhang Y Li YR Strain induced magnetic anisotropy in highly epitaxial CoFe_2_O_4_ thin films Appl Phys Lett 2006 89 262506 10.1063/1.2424444

[B13] Comes R Liu H Khokhlov M Kasica R Lu J Wolf SA Directed self-assembly of epitaxial CoFe_2_O_4_-BiFeO_3_ multiferroic nanocomposites Nano Lett 2012 12 2367 2373 10.1021/nl3003396 22486737

[B14] Ballav N Schilp S Zharnikov M Electron-beam chemical lithography with aliphatic self-assembled monolayers Angew Chem 2008 120 1443 1446 10.1002/ange.200704105 18188856

[B15] Tseng AA Recent developments in micromilling using focused ion beam technology J Micromech Microeng 2004 14 R15 R34 10.1088/0960-1317/14/4/R01

[B16] Nam CY Tham D Fischer JE Disorder effects in focused-ion-beam-deposited Pt contacts on GaN nanowires Nano Lett 2005 5 2029 2033 10.1021/nl0515697 16218732

[B17] Narayanan TN Mandal BP Tyagi AK Kumarasiri A Zhan X Hahm MG Anantharaman MR Lawes G Ajayan PM Hybrid multiferroic nanostructure with magnetic-dielectric coupling Nano Lett 2012 12 3025 3030 10.1021/nl300849u 22545916

[B18] Moyen E Santinacci L Masson L Wulfhekel W Hanbucken M A novel self-ordered sub-10 nm nanopore template for nanotechnology Adv Mater 2012 24 5094 5098 10.1002/adma.201200648 22718558

[B19] Xiao ZL Han CY Welp U Wang HH Vlasko-Vlasov VK Kwok WK Miller DJ Hiller JM Cook RE Willing GA Crabtree GW Nickel antidot arrays on anodic alumina substrates Appl Phys Lett 2002 81 2869 10.1063/1.1512993

[B20] Hu W Chen X Wu G Lin Y Qin N Bao D Bipolar and tri-state unipolar resistive switching behaviors in Ag/ZnFe_2_O_4_/Pt memory devices Appl Phys Lett 2012 101 063501 10.1063/1.4744950

[B21] Luo JM Lin SP Zheng Y Wang B Nonpolar resistive switching in Mn-doped BiFeO_3_ thin films by chemical solution deposition Appl Phys Lett 2012 101 062902 10.1063/1.4742897

